# Comparing Loneliness Among Individuals in Long-Term Care Settings and the Community

**DOI:** 10.1093/geroni/igab046.2214

**Published:** 2021-12-17

**Authors:** Cassandra Hua

**Affiliations:** Brown University, Providence, Rhode Island, United States

## Abstract

We used the NHATS COVID-19 module to examine whether individuals in long-term care communities were lonelier than individuals in the community during the pandemic. Additionally, we examined whether individuals in long-term care communities with more restrictive policies concerning visitors and communal activities were more likely to experience loneliness than individuals in communities with less restrictive policies. Approximately 45% of individuals in long-term care communities (n=134) felt at least a moderate amount of loneliness during COVID-19 when compared to 34% of individuals in the community (n= 2,666) (p<.05). However, the association was no longer statistically significant after adjusting for age, race, and sex. Among individuals in long-term care communities with the most restrictive policies, 48% experienced loneliness compared to 44% individuals in less restrictive communities. However, this finding was not statistically significant. Discussion will focus on similarities and differences within these populations that could have led to these results.

